# Large Vessel Occlusion Stroke Detection in the Prehospital Environment

**DOI:** 10.1007/s40138-021-00234-9

**Published:** 2021-06-28

**Authors:** Lauren Patrick, Wade Smith, Kevin J. Keenan

**Affiliations:** 1 University of California San Francisco, 505 Parnassus Ave, S784, San Francisco, CA 94122, USA; 2 University of California Davis, Davis, USA

**Keywords:** Large vessel occlusion, Prehospital stroke triage, Acute ischemic stroke, Stroke scale, Medical devices for stroke detection, Mobile stroke units, LVO device

## Abstract

**Purpose of Review:**

Endovascular therapy for acute ischemic stroke secondary to large vessel occlusion (LVO) is time-dependent. Prehospital patients with suspected LVO stroke should be triaged directly to specialized stroke centers for endovascular therapy. This review describes advances in LVO detection among prehospital suspected stroke patients.

**Recent Findings:**

Clinical prehospital stroke severity tools have been validated in the prehospital setting. Devices including EEG, SSEPs, TCD, cranial accelerometry, and volumetric impedance phase-shift-spectroscopy have recently published data regarding LVO detection in hospital settings. Mobile stroke units bring thrombolysis and vessel imaging to patients.

**Summary:**

The use of a prehospital stroke severity tool for LVO triage is now widely supported. Ease of use should be prioritized as there are no meaningful differences in diagnostic performance amongst tools. LVO diagnostic devices are promising, but none have been validated in the prehospital setting. Mobile stroke units improve patient outcomes and cost-effectiveness analyses are underway.

## Introduction

Endovascular therapy (EVT) using mechanical thrombectomy with or without intravenous thrombolysis (IVT) has been proven superior to standard medical care, including IVT alone for patients with acute ischemic stroke secondary to large vessel occlusion (LVO) [1, 2]. More recent trials have demonstrated that extended window thrombectomy (between 6 and 24 h) guided by acute CT or MRI perfusion imaging is safe and highly effective in the presence of a small to medium-sized infarct core [3•, 4, 5•]. Though strong collateral blood flow enables some patients with slowly progressing infarcts to remain EVT candidates even 24 hours after onset, it is not currently possible to identify which patients will instead suffer early infarction, and faster reperfusion with better outcomes [6–8]. The “time is brain” mantra, which describes the time-dependent loss of brain tissue as the untreated stroke progresses, remains a central tenet of prehospital stroke care [9–11].

In 2021, a multi-society consensus statement from leading stroke and emergency medical services (EMS) experts recommended that regional stroke destination plans prioritize the transportation of suspected LVO patients to Comprehensive Stroke Centers (CSC) for emergent EVT when within acceptable transportation times [10]. CSCs provide the highest level of stroke care and must be IVT and EVT-capable at all times. As of 2019, there were less than 300 CSC in the USA. In contrast, more common Primary Stroke Centers (PSCs) provide IVT but are not required to be EVT-capable. A third designation, Thrombectomy-capable Stroke Centers (TSCs), has been created for centers that provide all PSC services and EVT but do not meet all CSC requirements. The requirement that CSCs and TSCs provide EVT and the potential for some PSCs to provide it as well complicates the terminology of LVO stroke triage destination plans. All other things being equal, including prehospital travel time, transportation to a CSC is preferred [10]. The terms EVT center and non-EVT center provide a distinction based on the goal of prehospital stroke destination plans: facilitating direct transportation of suspected LVO patients to EVT centers.

EMS transportation of IVT and EVT eligible LVO stroke patients to a closer non-EVT center may facilitate faster IVT; however, IVT recanalization rates are low for LVO stroke, and interfacility transfers for EVT are associated with treatment delays and worse outcomes [12–15]. In some cases, transfer delays may preclude EVT altogether [14]. Accurate prehospital LVO identification would enable EMS providers to triage LVO stroke patients directly to EVT centers, potentially bypassing closer non-EVT centers, and avoid interfacility transfers [16, 17]. Prehospital triage of suspected LVO stroke patients should facilitate fast EVT treatment times and improve stroke outcomes, though we currently do not have prospective randomized studies demonstrating this. Additional potential benefits of LVO identification tools include pre-notification of EVT-capable centers of a patient’s impending arrival so that EVT teams can prepare and aiding emergency departments without CT angiography (CTA) capabilities in transfer decisions for patients with suspected LVO [18]. Potential disadvantages of using prehospital LVO identification tools to bypass a nearby non-EVT center in favor of a more distant EVT center include the possibility of missing opportunities for IVT for patients nearing the end of the standard time window, additional EMS transportation time, concerns that false positives will saturate CSCs with patients that do not have LVOs, and separation of patients from their support structure [18].

This review details the use of varying methods of LVO identification in patients with suspected stroke, including updates in clinical prehospital stroke scales performed by EMS personnel, various portable medical devices, and mobile stroke units.

## Prehospital Stroke Severity Tools

The American Heart Association (AHA)/American Stroke Association (ASA) provides the Mission Lifeline: Stroke EMS Acute Stroke Routing Algorithm as a model for prehospital LVO triage using currently available prehospital stroke scales [19]. The first test in this triage algorithm is a validated prehospital stroke identification screen that, combined with EMS suspicion of stroke, is intended to identify prehospital suspected stroke patients in general. These screens are typically scored as either positive or negative. They include the Cincinnati Prehospital Stroke Scale (CPSS), Los Angeles Prehospital Stroke Screen (LAPSS), Melbourne Ambulance Stroke Screen (MASS), and the Recognition of Stroke in the Emergency Room (ROSIER) Scale [20–23]. The items of the CPSS are sometimes referred to as the Face-Arm-Speech-Time (FAST) test. EMS providers assess patients with positive screens using one of many currently available validated stroke severity tools to identify patients with suspected LVO stroke. Sometimes referred to as LVO stroke scales, stroke severity tools typically contain various combinations of items from the neurological examination that are highly predictive of LVO. Patients with positive tests, compatible IVT or EVT treatment time windows, and EVT centers within travel time limitations are then triaged directly to EVT centers in hopes of expediting EVT for those confirmed to have LVO stroke on arrival. Patients with negative tests are transported to the nearest stroke center.

With consensus statements and triage algorithms now available, the remaining question is which validated stroke severity tool to use. Overall, the data quality has dramatically improved since the 2018 systematic review that determined there is insufficient evidence to conclude that any single severity tool is better than the others [24]. This is largely due to two large prospective observational studies from the Netherlands by Duvekot et al. and Nguyen et al. that compared the performance of multiple stroke severity tools when used by EMS providers taking care of prehospital suspected stroke patients [25•, 26]. However, the answer remains the same: prehospital stroke severity tools perform similarly. At standard cutpoints, they have poor to moderate sensitivity (38–67%) and moderate to high specificity (80–93%). None achieve sensitivity and specificity ≥ 80% simultaneously. It was notable that standard stroke severity tool score cutpoints (often those proposed in their original studies) are more specific than sensitive in these prehospital validation studies. This could be due to the low prevalence of LVO in these studies (7.9% and 12%) despite the inclusion of more distal LVOs.

The ACT-FAST Stroke Algorithm created by Zhao et al. has also been validated in the prehospital setting and includes a unique treatment eligibility screen step. The algorithm provides a binary positive or negative result with relatively high and balanced sensitivity (75.8%) and specificity (81.8%) when using an extended definition of LVO that includes proximal LVOs plus M2s, P1s, and symptomatic stenoses. However, direct comparisons with the severity tools prospectively and simultaneously evaluated in the studies above are difficult because ACT-FAST was omitted. Most striking is the difference in positive predictive values (PPVs), which exceeded 50% for the ACT-FAST extended LVO definition. In comparison, most severity scores studied by Nguyen et al. in the Netherlands had PPVs in the 20% range [26]. Differences in LVO prevalence likely contribute as higher prevalence facilitates higher PPVs [27]. When ICA, M1, and M2 occlusions are included in the definition, the data available to date suggest the prevalence of LVO stroke among prehospital suspected stroke patients is between 4 and 15% [25, 26, 28–30]. This has practical implications for EMS systems and researchers planning future trials as PPVs for LVO alone are not likely to reach 60% or higher unless a region has an especially high prevalence or LVO stroke identification methods improve (Table 1). Adding intracerebral hemorrhage (ICH) to LVO when defining true severity tool positives further increases PPVs. This is a reasonable approach given that severe ICH patients are often transferred to higher levels of care.

Given the similarities in stroke severity tool performance and goal of widespread adoption by EMS providers, ease of use is a reasonable consideration when choosing among them. The easiest approach in the USA would be to change the CPSS, which is currently used as a positive or negative prehospital stroke identification screen, into a stroke severity tool with scores ranging from 0 to 3 [23, 31]. There are many other reasonable options (Table 1). The Los Angeles Motor Scale (LAMS) focuses on face, arm, and grip weakness, thereby avoiding more complicated tests for cortical symptoms [32]. G-FAST adds gaze deviation, the neurological examination finding most predictive of LVO, to the CPSS [33, 34]. C-STAT was designed to be used without dedicated training, and ACT-FAST uses an 8-min training video and practical guidance that simplifies assessments for aphasia, gaze deviation, and neglect [35, 36]. Though RACE scoring is more complicated than other severity tools, multiple studies have validated its diagnostic performance when used by EMS providers [37–40].

With so many reasonable choices, our focus should shift from which severity tool to use to clarifying our diagnostic and patient care priorities. Most prehospital stroke severity tools have multiple possible scores and choosing a lower score cutpoint would allow for higher sensitivity (not missing an LVO treatment opportunity) to be prioritized over specificity. More false positives would accompany this shift in priorities because these tools are not simultaneously sensitive and specific. However, it is important to prioritize sensitivity in highly morbid diseases with time-sensitive and profoundly effective interventions like EVT for LVO stroke.

This is not as daunting as it may seem. Multiple modeling studies have found that LVO triage may not result in overwhelmed stroke centers or missed thrombolysis opportunities [13, 30, 36, 41]. If sensitivity is prioritized, CPSS ≥ 2 would be a strong and easy stroke severity tool choice because it performs just as well as other tools at their sensitive score cutpoints (86% sensitivity and 61% specificity) and would require little to no additional EMS training [25].

Ultimately, prehospital stroke severity tools are limited by their sensitivity and specificity tradeoffs. Hopefully, the addition of the technologies discussed below will someday allow for highly sensitive and highly specific LVO triage that will expedite access to EVT while minimizing false positives.

## Medical Devices

There has been increasing interest and research in portable and non-invasive external diagnostic devices for prehospital LVO stroke identification and triage. We will briefly discuss various forms of biometric devices reported in the peer-reviewed literature, including electroencephalography with or without somatosensory evoked potentials, transcranial Doppler, cranial accelerometry, and volumetric impedance phase-shift spectroscopy (Table 2).

### Electroencephalography and Somatosensory Evoked Potentials

Electroencephalography (EEG) analyzes normal and abnormal brain electrical activity by transducing electrical potential differences on the patient’s scalp. EEG is primarily used to diagnose seizures and manage epilepsy. It has been used to monitor cerebral ischemia, particularly in the intraoperative setting during carotid artery surgery and, more recently, in acute ischemic stroke [42]. EEG can detect early brain function changes following ischemia and is sensitive for detecting early stroke. Until recently, its clinical application has been limited by a cumbersome electrode application process that requires technical expertise. This is mitigated somewhat with dry-electrodes [43, 44]. Erani et al. evaluated 100 patients presenting to an emergency department with suspected acute stroke using a dry-electrode EEG system. Their data were used to create models that classified patients as having an acute ischemic stroke/TIA or not. The presence of LVO stroke or not was studied in a secondary analysis. They demonstrated that dry-EEG is feasible in the emergency department and found models combining the clinical examination and EEG data performed better than either data source alone [45]. One key limitation was that the median emergency department arrival to EEG recording time was 3.7 h. This introduces the potential for physiology to change between arrival and recording, but this will likely be overcome by prehospital studies.

A recent publication by Sergot and the EDGAR Study Group investigated a portable LVO-detection device (PLD) that used EEG combined with somatosensory evoked potentials (SSEPs) to identify LVO stroke in emergency departments [46•]. The study included patients presenting with NIHSS scores greater than one within 24 h of last seen normal. Its multi-center design and assessments of user-rated device usability were two unique strengths of this study. The LVO stroke model derived from the data was simultaneously sensitive and specific (80% each), novice operators could apply the device quickly, and usability ratings were favorable. Limitations were similar to other device model derivation studies and included a small sample size (109) and convenience sampling. As the investigators note, future studies will need to validate the device in the prehospital setting with fewer exclusions of patients with comorbid neurological diseases, especially prior stroke with residual deficits.

### Transcranial Doppler

Transcranial Doppler (TCD) is a safe ultrasound-based method of evaluating cerebral hemodynamics and documenting blood flow in the middle cerebral artery. A pulsed Doppler ultrasound transducer is used to assess intracerebral blood flow through cranial “windows” [47]. A systematic review of diagnostic utility of TCD in the evaluation of patients with acute ischemic stroke secondary to LVO demonstrated findings such as diminished flow and asymmetry indices were shown to be suggestive of large vessel occlusion with a sensitivity of 68–100% and specificity of 78–99% [48]. Its need for expert analysis, user dependency, equipment cost, and insufficient acoustic windows in approximately 10% of patients have limited its utilization in detecting acute ischemic stroke [49, 50]. However, there have been recent strides regarding complex training requirements of TCD operators and approach to results analysis. Cerebral blood flow velocity waveforms have been categorized using the Thrombolysis in Brain Ischemia (TIBI) grading system which requires expert analysis, so TCD-derived morphological biomarkers such as Velocity Asymmetry Index (VAI) and Velocity Curvature Index (VCI) have been studied as user-independent LVO metrics that may lead to increased TCD use in the prehospital setting [51–55]. A robotically assisted ultrasound system has recently been developed to evaluate brain health in the acute setting with machine learning analysis of data [56]. To date, there is no peer-reviewed data regarding robotically assisted TCD, though initial results presented at a Society of Vascular and Interventional Neurology workshop in 2017 were promising [56].

### Cranial Accelerometry

Cranial accelerometry is used to measure the headpulse and has been investigated as a tool to diagnose LVO stroke by the authors of this review. Headpulse refers to nearly imperceptible head movements with each cardiac contraction cycle in response to blood flow forces transmitted via the carotid and vertebral arteries. This is measured using accelerometers in contact with the skull combined with electrocardiogram leads. Patients with LVO stroke may have chaotic head pulse patterns that do not correlate with cardiac contraction cycles; this is hypothesized to be due to the obstruction of blood flow on one side by the LVO. Cranial accelerometry alone (i.e., not considering any neurological examination features) was 73% sensitive and 87% specific for LVO in an initial study [57]. In a subsequent study, Keenan et al. combined features of the neurological examination with cranial accelerometry from a larger but overlapping cohort of 68 patients [58]. The study included patients from the community undergoing stroke alerts on arrival and EVT candidates transferred with a known diagnosis of LVO from outside hospitals. Recordings were obtained as soon as possible after initial stroke imaging and before EVT when applicable. LVO detection using cranial accelerometry alone was 65% sensitive and 87% specific. In an exploratory analysis, sensitivity and specificity improved to 91% and 93% respectively by first classifying patients without asymmetric arm weakness as negative, then training a cranial accelerometry model to use the remaining 35 patients with asymmetric arm weakness. The small sample size, convenience sampling, and exploratory nature of these findings put this study at risk for overfitting. External validation and prehospital feasibility studies are currently underway.

### Volumetric Impedance Phase-Shift Spectroscopy

Volumetric impedance phase-shift spectroscopy (VIPS) technology involves passing low-power electromagnetic waves through the brain to detect asymmetries and electrolyte concentration changes [47]. A device that utilizes this technology via a “halo-like” visor that sits on the patient’s head has been studied in traumatic brain injury given its high sensitivity for brain edema. The VITAL study tested a portable VIPS device’s diagnostic capability in a pooled population of patients (248 total) presenting with suspected stroke at a comprehensive medical center, brain pathology without suspected stroke, and in healthy volunteers [59]. The investigators created a model that distinguished “severe stroke” which included LVO stroke, very large ICH (>60mL), and severe arterial stenosis from minor stroke and other brain pathologies with 93% sensitivity and 92% specificity. Specificity was 87% when tested using the entire sample, including those without brain pathology. The larger sample size is a strength of this study; however, it is unclear if the addition of healthy volunteers and non-suspected stroke brain pathologies will limit generalizability to LVO triage. Limitations were otherwise similar to the studies above. External validation and prehospital studies are required.

### Future LVO Device Studies

At present, none of the LVO devices in development have reported the results of external validation, prehospital feasibility, or diagnostic accuracy studies in ambulances with suspected stroke patients. As investigators design these studies, it will be critical to follow the Standards for Reporting of Diagnostic Accuracy Studies (STARD) guidelines. Many peer-review journals require completion of the STARD checklist during submission, and more importantly, adherence to their guidance will reduce the impact of avoidable biases, increase transparency, and enable researchers to draw meaningful conclusions from new studies as the field advances [60].

## Mobile Stroke Units

The concept of mobile stroke units (MSUs) was published in 2003 by Fassbender to “bring treatment to the patient, rather than the patient to the treatment” and was first established in Germany in 2008 [61]. MSUs typically contain the following components: standard ambulance equipment and medications, point-of-care lab equipment, CT scanner (with recent versions capable of performing CTA), CT technologist, and a physician (in-person or via telemedicine) [62]. MSUs are a potentially significant solution to increasing stroke patient access to thrombolysis, especially in rural areas. MSUs that perform CTA can also facilitate the transfer of patients with LVOs to EVT centers. Multiple studies have shown that MSUs can significantly reduce the time to thrombolytic treatment and significantly increase the number of patients treated within the first 60 min since last seen normal time [62].

A recent publication by Ebinger and colleagues in Berlin demonstrated an association between MSUs and improved functional outcomes in acute ischemic stroke patients [63]. They conducted a prospective, nonrandomized controlled intervention study in which both a conventional ambulance and MSU were dispatched to emergency calls suspicious for acute stroke. Functional outcomes of patients discharged with a final diagnosis of acute stroke eligible for thrombolysis and/or thrombectomy were analyzed, with a primary outcome of modified Rankin Score at 3 months. Overall, the dispatch of MSUs compared to conventional ambulances alone was associated with significantly lower global disability. Limitations of the study include lack of randomization (patients were allocated to MSU or conventional ambulance based on the availability of MSUs), and as the authors note, they did not assess the outcomes of patients with discharge diagnoses other than ischemic stroke or transient ischemic attack.

The BEST-MSU study is the first multicenter randomized controlled trial comparing standard ambulance care versus MSU care, including prehospital thrombolysis. It began in 2014 in Texas and has demonstrated the safety and feasibility of MSUs, the reliability of telemedicine technology, as well as faster door-to-groin puncture times for patients requiring thrombectomy [64]. Results were presented in abstract form at the 2021 International Stroke Conference and suggested that MSU care resulted in significantly better clinical outcomes than standard care. It was estimated that for every 100 patients treated with an MSU 27 more will have less disability and 11 more will be disability-free at follow-up [65•]. We eagerly anticipate the upcoming peer-reviewed manuscript. Cost-effectiveness, implementation, and generalizability studies as well as pathways to clinical care reimbursement are key next steps that will impact the proliferation of this innovative approach [66]. Where available, the ability of MSUs to perform CTA in the prehospital setting will facilitate LVO triage without the false-positive and false-negative concerns inherent to stroke severity tools or LVO devices.

## Conclusions

Prehospital triage of patients with suspected LVO to EVT centers is now recommended. The Mission Lifeline: Stroke EMS Triage Algorithm provides a framework that includes neurological examination-based prehospital stroke severity tools. Decisions regarding which severity to tool to use can likely be based on ease of use as prospective comparative studies did not demonstrate meaningful performance differences. Standard score cutpoints of many of these tools favor specificity over sensitivity, which will lead to many missed LVO strokes. Sensitivity should be prioritized, and CPSS ≥ 2 provides similar performance compared to other sensitive stroke severity tool cutpoints but would not require significant training to implement. Several LVO stroke diagnostic devices have published peer-review initial studies, but each device will require prehospital validation before becoming available for use. Mobile stroke units improve patient outcomes and, when available, can perform vessel imaging on scene to diagnose or rule out LVO stroke. Cost-effectiveness analyses are planned. Ongoing improvements in prehospital LVO triage will likely require tailoring the diagnostic approach to the needs of each region.

## Figures and Tables

**Table 1:** Summary of prehospital stroke severity tools

	ACT-FAST^1^	CG-FAST^2^	CPSS^2^	C-STAT^2^	FAST-ED^3^	FAST-PLUS^2^	G-FAST^2^	LAMS^2^	PASS^2^	RACE^2^

No. of items assessed	3	5	3	3	5	4	4	3	3	6
Face weakness		x	x		x	x	x	x		x
Arm weakness	x	x	x	x	x	x	x	x	x	x
Leg weakness						x				x
Grip strength								x		
Gaze deviation		x		x	x		x		x	x
Hemineglect	x				x					x
Dysarthria or Aphasia	x*	x	x		x	x	x			x
LOC questions		x		x					x	
Standard threshold for a positive test	+/−	≥4	=3	≥2	≥4	+/−	≥3	≥4	≥2	≥5
Sensitivity◆	0.76	0.50	0.57	0.50	0.60	0.60	0.67	0.63	0.59	0.67
Specificity◆	0.82	0.89	0.85	0.85	0.85	0.83	0.82	0.84	0.83	0.87
Probability of LVO after a positive test (PPV)◆ with a baseline:										
5% LVO prevalence	18%	19%	17%	15%	17%	16%	16%	17%	15%	21%
10% LVO prevalence	32%	34%	30%	27%	31%	28%	29%	30%	28%	36%
15% LVO prevalence	43%	45%	40%	37%	41%	38%	40%	41%	38%	48%
20% LVO prevalence	51%	53%	49%	45%	50%	47%	48%	50%	46%	56%

*ACT-FAST*, Ambulance Clinical Triage For Acute Stroke Treatment, *CG-FAST* Conveniently-Grasped Field Assessment Stroke Triage, *CPSS* Cincinnati Prehospital Stroke Scale, *C-STAT* Cincinnati Stroke Triage Assessment Tool, *FAST-ED* Field Assessment Stroke Triage for Emergency Destination, *FAST-PLUS* Face-Arm-Speech-Time plus severe arm or leg motor deficit, *G-FAST* Gaze-Face-Arm-Speech-Time, *LAMS* Los Angeles Motor Scale, *PASS* Prehospital Acute Stroke Severity, *RACE* Rapid Arterial oCclusion Evaluation, *LOC* level of consciousness, *LVO* large vessel occlusion, *PPV* positive predictive value, +/− scored as either positive or negative

* ACT-FAST tests for language difficulty and isolated dysarthria does not count

◆ Sensitivity, specificity, and PPV are reported at each severity tool’s standard threshold. For tools with multiple possible thresholds, lower thresholds will be more sensitive but less specific. Higher thresholds will be less sensitive but more specific

1 Sensitivity and specificity as noted in Zhao et al.’s study (ref. 36)

2 Sensitivity and specificity as noted in Duvekot et al.’s study (ref. 25)

3 Sensitivity and specificity as noted in Nguyen et al.’s study (ref. 26)

**Table 2:** Non-invasive biometric devices for large vessel occlusion detection

Technology	Description

Electroencephalography (EEG)	Electrodes adhered to scalp monitor cerebral electrical activity
Somatosensory evoked potentials (SSEP)	Electrical signals in the brain generated by sensory stimuli in a peripheral nerve; sensory cortex is supplied by middle cerebral artery
Transcranial Doppler (TCD)	Monitors cerebral hemodynamics via pulsed Doppler ultrasound transducer to assess intracranial blood flow
Cranial accelerometry	Subtle head oscillations associated with cardiac contraction measured by accelerometers on headband and electrocardiogram leads
Volumetric impedance phase-shift spectroscopy	Passage of low-power electromagnetic waves through the brain to detect asymmetries in electrical properties
